# Phytoplankton communities determine the spatio-temporal heterogeneity of alkaline phosphatase activity: evidence from a tributary of the Three Gorges Reservoir

**DOI:** 10.1038/s41598-017-16740-4

**Published:** 2017-11-27

**Authors:** Yijun Yuan, Yonghong Bi, Zhengyu Hu

**Affiliations:** 1 State Key Laboratory of Freshwater Ecology and Biotechnology, Institute of Hydrobiology, Chinese Academy of Sciences, 430072 Wuhan, China; 2 Key Laboratory of Nuclear Resources and Environment, East China University of Technology, 330013 Nanchang, China; 3 University of Chinese Academy of Sciences, 100049 Beijing, China

## Abstract

In order to reveal the role of phytoplankton in the spatio-temporal distribution of alkaline phosphatase activity (APA), monthly investigations were conducted in the Xiaojiang River, a tributary of the Three Gorges Reservoir in China. Different APA fractions, environmental parameters, and phytoplankton communities were followed. High spatio-temporal variations of APA were observed, with the highest value in summer and the lowest in winter. The annual average APA_T_ (total alkaline phosphatase activity) ranged from 7.78–14.03 nmol∙L^−1^∙min^−1^ with the highest in the midstream and the lowest in the estuary. The dominant phytoplankton phyla in summer and winter were *Cyanophyta* and *Bacillariophyta*, respectively. The mean cell density in the midstream and in the estuary was 5.2 × 10^7^ cell∙L^−1^ and 1.4 × 10^7^ cell∙L^−1^, respectively. That APA_>3.0 μm_ was significantly higher than APA_0.45-3 μm_ indicating phytoplankton was the main contributor to alkaline phosphatase. Correlation analysis indicated the dominant species and cell density could determine the distribution pattern of APA. Turbidity, total phosphorus, chemical oxygen demand, water temperature (WT), pH and chlorophyll *a* were proved to be positively correlated with APA; soluble reactive phosphorus, conductivity, transparency and water level(WL) were negatively correlated with APA. It was concluded that spatio-temporal heterogeneity of APA determined by phytoplankton communities was related to WT and WL.

## Introduction

Phosphorus is always treated as a limiting nutrient in many freshwater ecosystems because it frequently limits the primary production^1,2^. The availability of phosphorus (P) is regarded as a crucial factor regulating the dynamic of phytoplankton *in situ*. There is no doubt that there are many types of phosphorus in waters, including the organic and inorganic phosphorus. Among the various forms of phosphorus, orthophosphate (o-P) or available soluble reactive phosphorus (SRP), the bioavailable form of phosphorus, can be depleted rapidly in freshwaters due to the rapid uptake^3^, which tends to limit algal growth in freshwater ecosystems. So the low concentrations of SRP can modify the structure of plankton communities and constrain phytoplankton distribution^4,5^. Some phytoplankton species, zooplankton and bacterioplankton can produce extracellular phosphatase liberating SRP from dissolved organic P compounds, which is one mechanism allowing the living organisms to overcome P limitation^6,7^.

Relationship between APA and phytoplankton has been paid more attention since 1960s^8,9^. Kalinowska tried to figure out the major contributor of APase through membrane filtration method^10^. Even if size fractionation by filtration is never completely absolute (i.e., overlapping size), it still provides useful insights on the major microorganisms possibly contributing to APA. The increase in APA can be attributed more to phytoplankton biomass than to the bacterial biomass^11^. Therefore, phytoplankton contributed greatly to APA production and was significantly influenced by P bioavailability. Production of extracellular phosphatases has been detected in many phytoplankton species^12–14^. Various taxa are exhibiting differences in the presence, localization and labeling pattern of phosphatases. Both seasonal and short-term variations also have been detected in enzyme activity of phytoplankton^15^. Enzyme-labeled fluorescence (ELF) analysis revealed pronounced differences in the makeup of phytoplankton responsible for APA in San Francisco and Monterey bays^16^.

The Three Gorges Reservoir (TGR) is the biggest deep river-type reservoir in the world. More than 170 tributaries carry runoff and bring nutrients and pollutants into it, which affected the trophic status and resulted in water blooms in some tributaries of the TGR^17^. Though many studies have been conducted to screen APase in different water bodies, little knowledge was obtained in the Three Gorges Reservoir (TGR). Due to the complicated relationship between APA and ecological factors, it is necessary to screen the distribution pattern of APA and its influencing factors in the TGR. Xiaojiang River is one of the tributaries in the TGR, which is suffered from water blooms frequently. Eutrophication in Xiaojiang River is very serious after the Three Gorges Dam (TGD)’s impoundment since 2003^18^. In this study, Xiaojiang River was selected as the delegate of the tributary in the TGR, phytoplankton and APA in Xiaojiang River were screened. Based on the related researches focused on the complicated relationship between APA and phytoplankton mentioned above, it was assumed that the phytoplankton community successions might lead to the spatio-temporal heterogeneity of alkaline phosphatase activity. In order to verify this hypothesis, monthly investigation was conducted, different APA fractions (APA_T_, APA_<0.45 μm_, APA_0.45-3 μm_ and APA_>3.0 μm_), environmental parameters and phytoplankton communities were screened synchronously. The role of phytoplankton communities in the spatio-temporal heterogeneity of APA and its influence factors in the Three Gorges Reservoir were demonstrated. The results of this study can help to know how APA production changes with phytoplankton communities’ successions in the TGR.

## Results

### APAT distribution pattern

**Figure 1:**
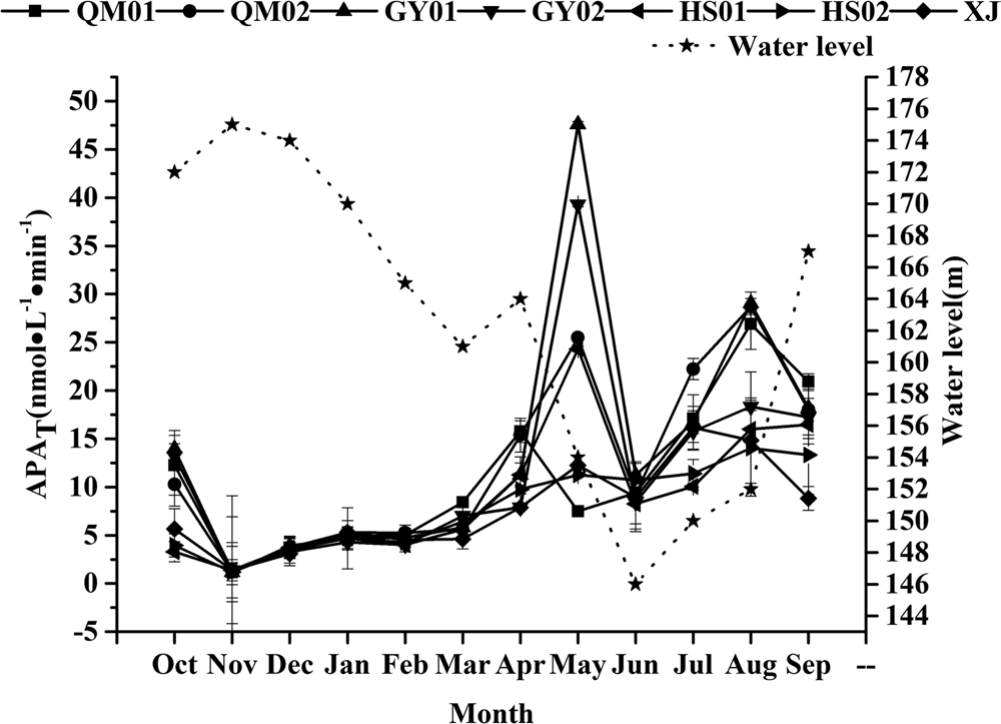
Monthly variations of the total APA concentrations in different sample sites and water level in the Xiaojiang River.

The APA_T_ ranged from 1.19–47.6 nmol·L^−1^·min^−1^ (Fig. 1). The lowest level of APA_T_ was observed in winter. Besides, the average APA_T_ in summer and autumn were higher than in other seasons. The mean water level was high in winter(169.7 ± 4.5 m) and low in summer(149.3 ± 3.1 m), the variations of water level presented different trends with that of APA_T_ at temporal scales.

The highest value of annual average APA_T_ in GY01 and lowest in XJ were also showed in Fig. 1. The average APA_T_ of GY01 in summer and autumn are higher than those of HS02 and XJ.

### Size-fractionation of APA

**Figure 2:**
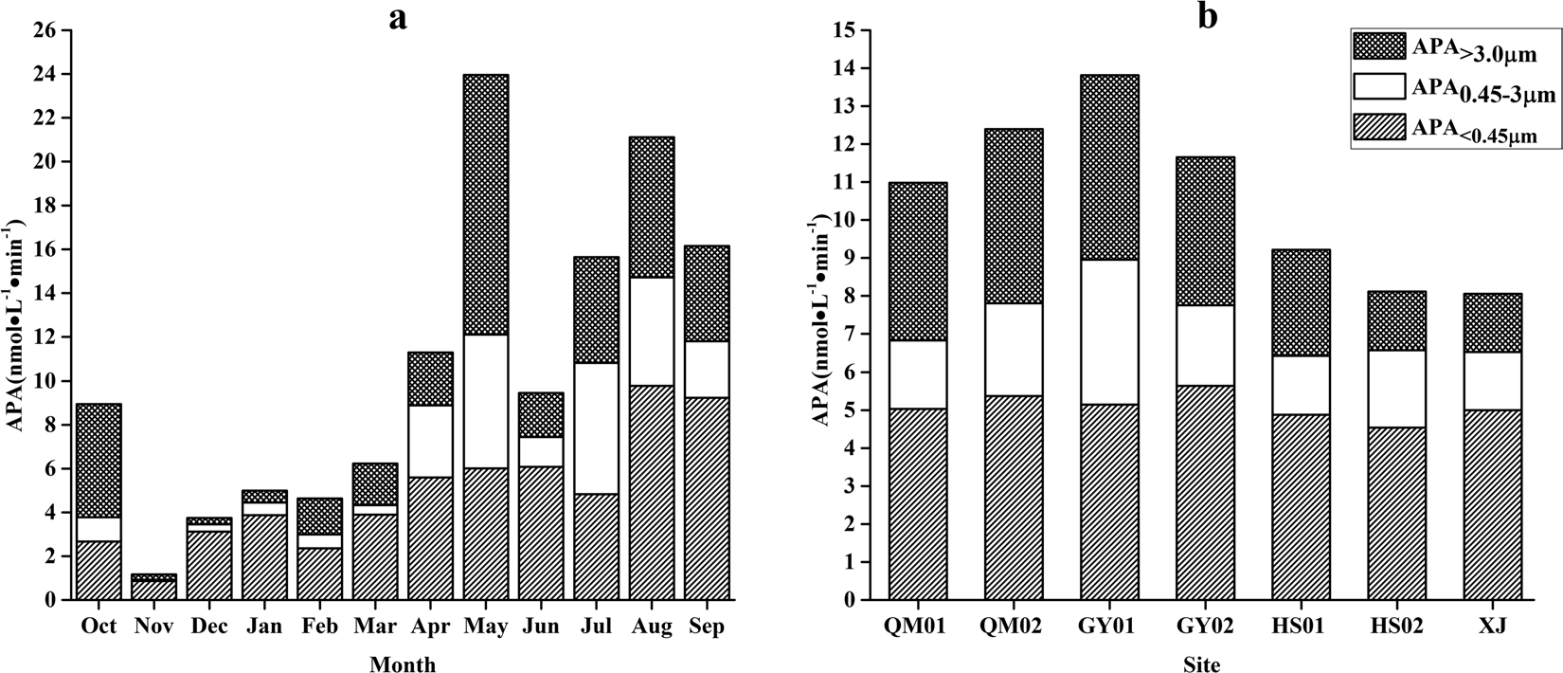
Seasonal (**a**) and spatial (**b**) variations of average size-fractionated APA in the Xiaojiang River. APA_>3.0 μm_: the alkaline phosphatase activity in algal fraction; APA_0.45-3.0 μm_: the alkaline phosphatase activity in bacterial fraction; APA_<0.45 μm_: picoplankton/dissolved alkaline phosphatase activity.

The average size-fractionated APA indicated that APA_<0.45 μm_ accounted for the major portion of APA_T_, whereas the average APA_>3.0 μm_ was higher than APA_0.45-3 μm_ (Fig. 2a). The average APA_>3.0 μm_ accounted for 28.1% of APA_T_ and APA_0.45-3 μm_ accounted for 16.7%. In addition, the size-fractionated APA (APA_<0.45 μm_, APA_0.45-3 μm_ and APA_>3.0 μm_) in summer and autumn are higher than those in winter.

At spatial scales, the average APA_T_ consisted of 30.2% APA_>3.0 μm_ and 20.4% APA_0.45-3 μm_ in all sites. The APA_<0.45 μm_ kept a relatively stable and high level. Both APA_0.45-3 μm_ and APA_>3.0 μm_ in midstream (GY01) are higher than those in estuary (XJ).

### Phytoplankton communities

**Figure 3:**
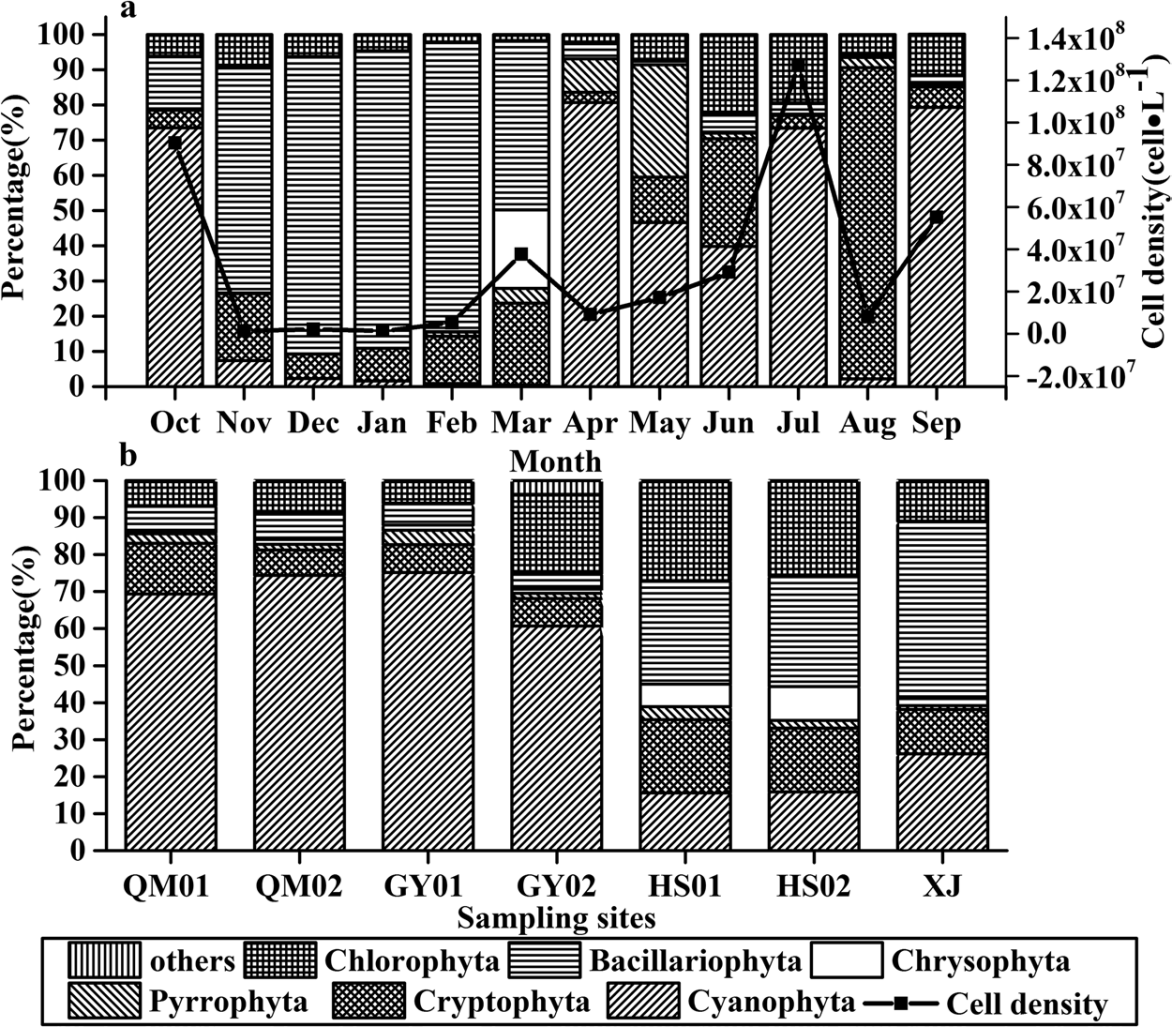
Seasonal (**a**) and spatial (**b**) variations of algal composition and algal cell density in the Xiaojiang River.

*Bacillariophyta* was the dominant group in winter and spring (72.7% of total cell density on average, Fig. 3a) except *cyanophyta* are dominant in April. In summer and autumn, phytoplankton mainly consisted of *Cyanophyta* (65.6% of total cell density on average) except the *Cryptophyta* accounted for 88.4% in August. The mean algal cells density was the highest in July 2014 (1.27 × 10^8^cell∙L^−1^), and the lowest in January 2014 (1.3 × 10^6^cell∙L^−1^). The cell density was higher in summer and autumn than in spring and winter. *Cyanophyta* dominated the phytoplankton in upstream (QM01, QM02, GY01, GY02, 69.9% of total cell density on average). In the downstream (HS01, HS02, XJ), phytoplankton mainly consisted of *Bacillariophyta* (35.3% of total cell density on average, Fig. 3b).

### Spatio-temporal characteristics of chlorophyll *a* and environmental parameters

**Figure 4:**
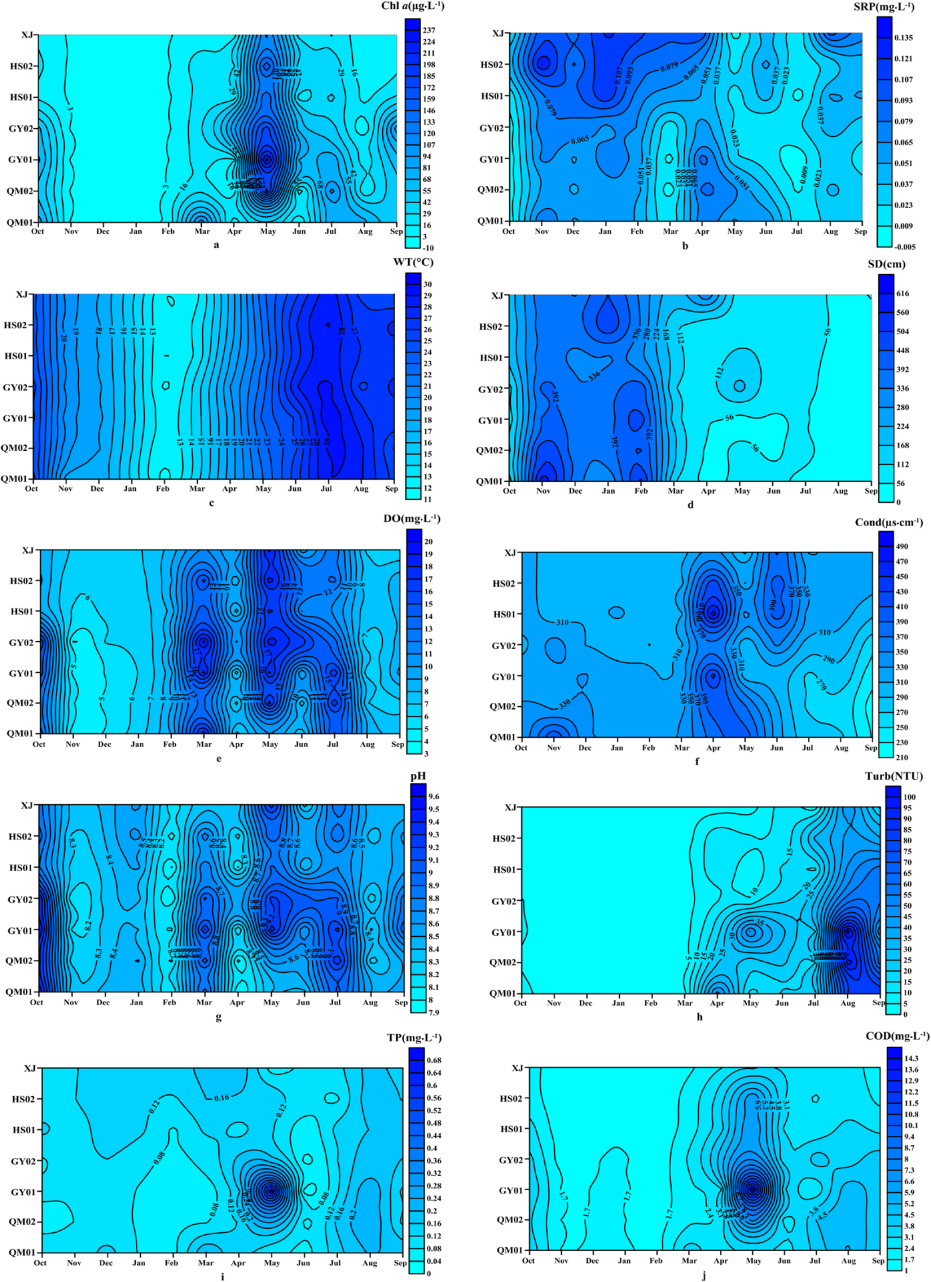
Temporal and spatial variations of a: chlorophyll (**a**) (Chl *a*) and other environmental parameters. (**b**) Soluble reactive phosphorus (SRP); (**c**) water temperature (WT); (**d**) transparency (SD); (**e**) dissolved oxygen (DO); (**f**) conductivity (Cond); (**g**) pH; (**h**) turbidity (Turb); (**i**) total phosphorus (TP) and (**j**) chemical oxygen demand (COD).

Spatio-temporal variations of chlorophyll *a* (Chl *a*) and environmental parameters could be observed in Fig. 4. The values of Chl *a*, total phosphorus (TP), chemical oxygen demand (COD) in spring were apparently higher than the values of other seasons, because the river suffered a *Microcystis* sp. bloom in May, which also resulted in the minimum values of SRP and transparency (SD) emerged. The levels of TP, COD, Chl *a*, water temperature (WT), turbidity (Turb), dissolved oxygen (DO) and pH stayed low in winter, contrary to the values of SRP and SD. The values of SRP fluctuated more frequently than other parameters in different seasons. At spatial scale, the concentrations of SRP and Chl *a* were higher in estuary than in upstream.

### Relationships between APA and environmental parameters

**Figure 5:**
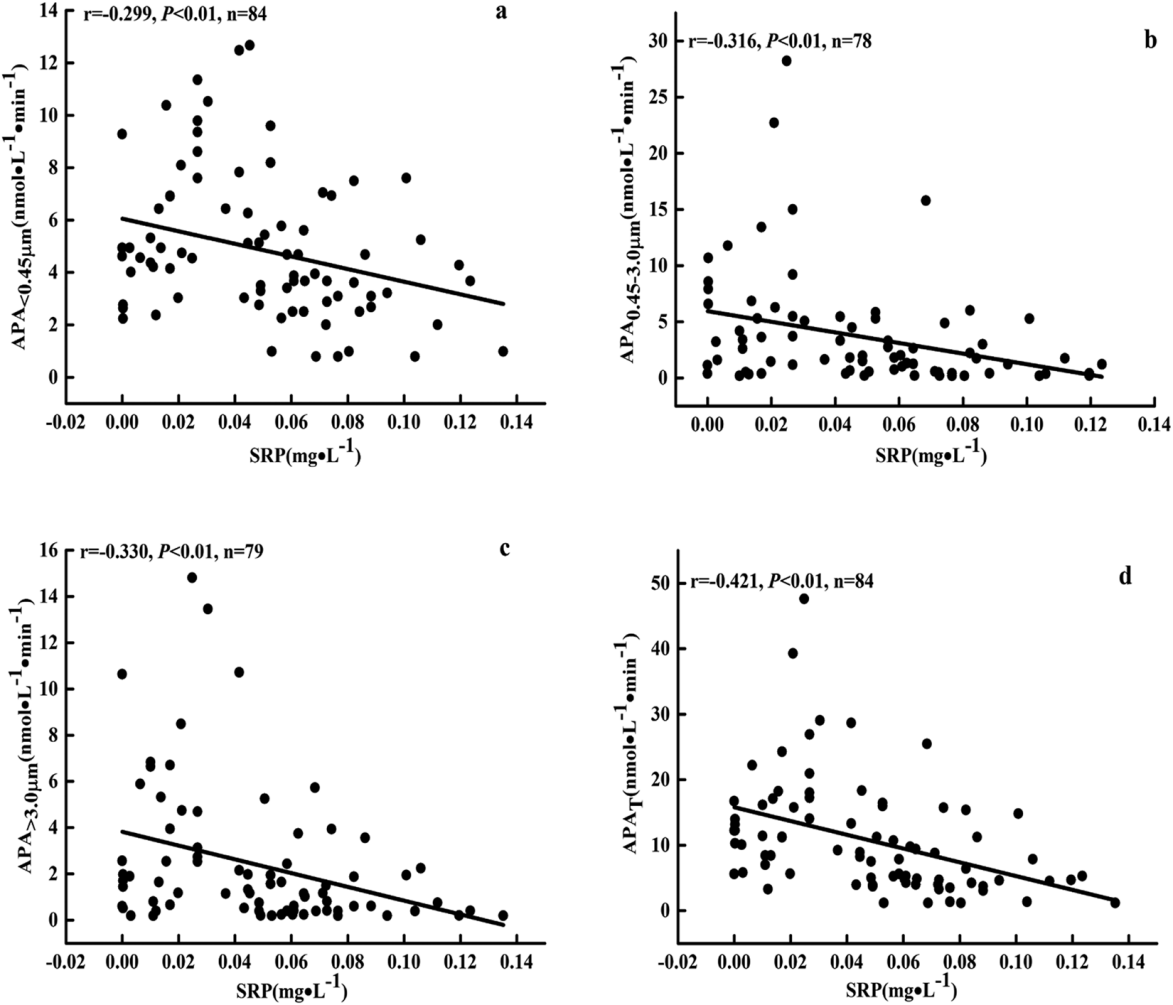
Relationship between soluble reactive phosphorus (SRP) concentrations and APA_<0.45 μm_ (**a**), APA_0.45-3 μm_ (**b**), APA_>3.0 μm_ (**c**) and APA_T_ (**d**) in the Xiaojiang River.

SRP concentrations showed negative correlation to APA_<0. 45 μm_ (Fig. 5a), APA_0.45-3 μm_ (Fig. 5b), APA_>3.0 μm_ (Fig. 5c) and APA_T_ (Fig. 5d). The Spearman correlations among environmental variables and APA_<0.45 μm_, APA_0.45-3 μm_, APA_>3.0 μm_ and APA_T_ were presented in Table 1. Turb, TP, COD, WT and pH were positively correlated with APA fractions. Cond., SD and WL were negatively correlated with APA.

**Table 1:** Spearman correlations between APA and 10 environmental variables: water temperature (WT); transparency (SD); dissolved oxygen (DO); conductivity (Cond); pH, turbidity (Turb); total phosphorus (TP); chemical oxygen demand (COD); water level (WL) in the Xiaojiang River.

	APA_T_(n = 84)	APA_<0.45 μm_(n = 84)	APA_>3.0 μm_(n = 78)	APA_0.45-3 μm_(n = 79)
WT	0.642**	0.562**	0.404**	0.609**
SD	−0.844**	−0.815**	−0.586**	−0.698**
DO	0.478**		0.382**	0.368**
Cond	−0.251*		−0.256*	
pH	0.405**		0.271*	0.271*
Turb	0.858**	0.834**	0.582**	0.753**
TP	0.388**	0.357**	0.346**	0.413**
COD	0.858**	0.684**	0.646**	0.751**
WL	−0.678*	−0.699*		−0.713**

*P < 0.05.

**P < 0.01.

**Figure 6:**
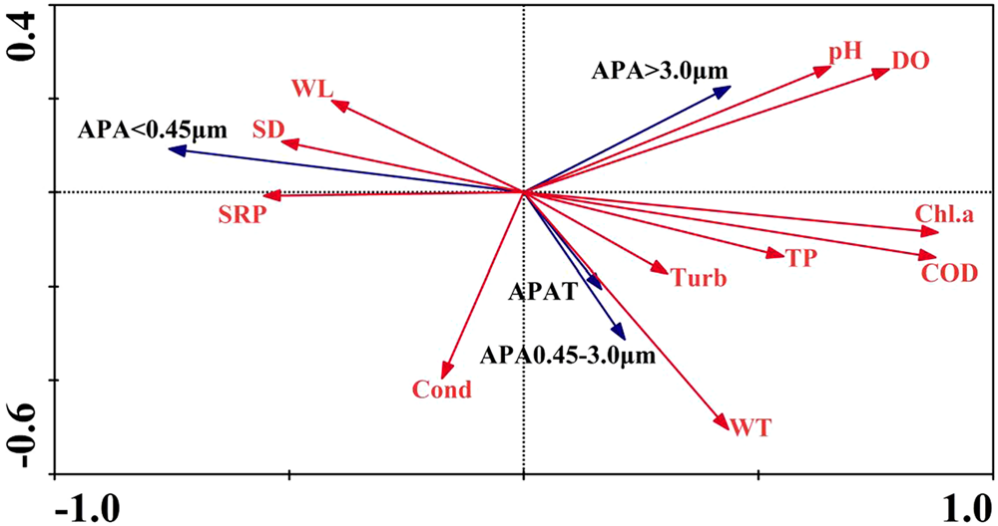
Biplot diagrams for RDA of the relationship between 11 environmental variables (red lines) and APA_<0.45 μm_, APA_>3.0 μm_, APA_0.45-3 μm_ (blue lines).

Redundancy analysis (RDA) was performed to analyze the relationship between environmental parameters and size-fractionated APA. The ordination diagrams of environmental variables and size-fractionated APA for axis 1 and axis 2 were shown in Fig. 6. The Monte Carlo test revealed that the first canonical axis and all canonical axes were significant (F = 25.932, P = 0.002; F = 3.086, P = 0.002; 499 random permutation). For environmental variables and size-fractionated APA, all canonical axes cumulatively explained 83.3% of the variance in APA–environment relationships, and the first two canonical axes accounted for 26.5% and 31.5% of the variance separately. The first axis was positively correlated with DO (0.57), COD (0.65) and negatively correlated with SRP (−0.41), SD (−0.38) and WL (−0.30). The second axis was mainly negatively correlated with Cond (−0.13) and WT (−0.16). APA_<0.45 μm_ and APA_>3.0 μm_ was the major portion of APA_T_. APA_>3.0 μm_ and APA_0.45-3 μm_ were located on the right-hand side of the biplot. They were correlated negatively with WL, SD, SRP and Cond, and positively with other parameters.

### Relationships between APA_>3.0 μm_ and algal cell density

**Figure 7:**
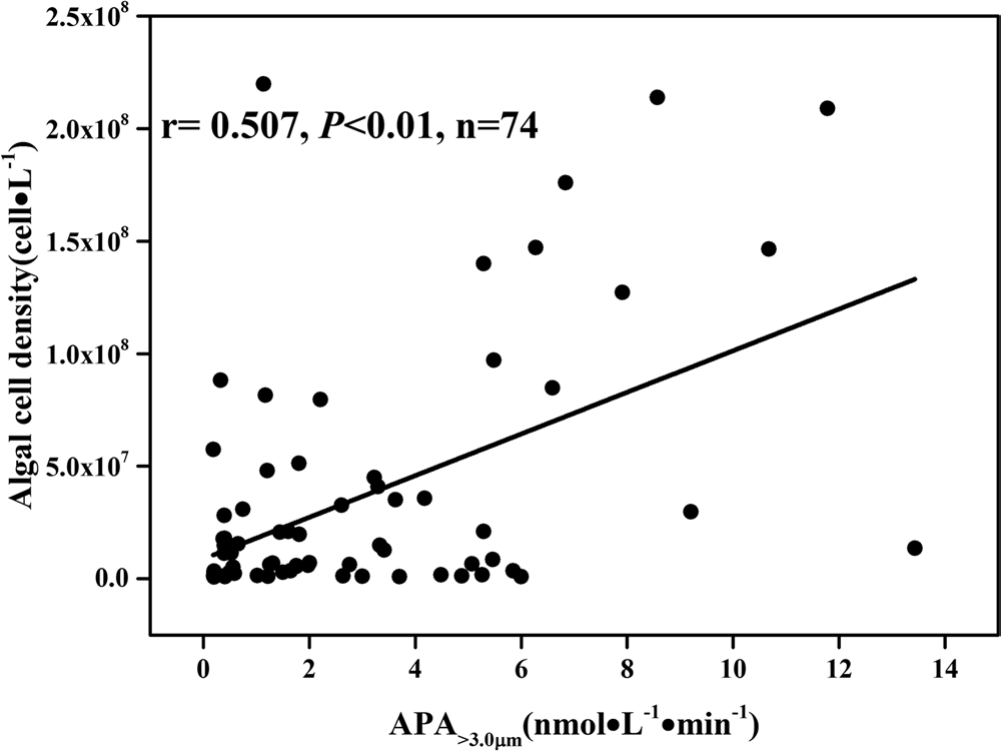
Relationships between APA_>3.0 μm_ and cell density in all sites.

APA_>3.0 μm_ reached the highest in midstream (GY01) in May (28.24 nmol∙L^−1^∙min^−1^), and undetectable in estuary (XJ) in December. Values ranged from 0.19–22.71 nmol∙L^−1^∙min^−1^at the other sites. The mean cell density was the highest in midstream (GY02, 5.2 × 10^7^cell∙L^−1^) and the lowest in estuary (XJ,1.4 × 10^7^cell∙L^−1^). A significant positive relationship was found between APA_>3.0 μm_ and cell density among all sites (Fig. 7).

## Discussion

APase has different sources, different kinds of bacteria, phytoplankton and zooplankton can excrete extracellular phosphatase^19^. Specific APA was related to different phosphatase producing organisms. In our investigation, APA_>3.0 μm_ contributed in average 28.1% in the APA_T_, while bacterial APA accounted for 16.7%, APA in algal fraction (APA_>3.0 μm_) was higher than that in the picoplankton/bacterial fraction (APA_0.45-3 μm_). Though many (not all) phytoplankton cells have a host heterotrophic bacteria inhabiting or in close association with cells, and the overlapping size on the filter also influenced the final data, which making it difficult to assign the different size fractionation by filtration to individual cells alone, it could be admitted that the coarser fraction (APA_>3.0 μm_), mainly from algae, was conventionally defined as “algal APA”^20^ due to phytoplankton was the main contributor according to its size, biomass and physiological activity. It was confirmed APA_>3.0 μm_ accounted for the major portion of total APA (55–87.9%) than APA_0.45-3 μm_^21^. It could be deduced that the phytoplankton was the major contributor of bulk APA based on the larger proportion of APA_>3.0 μm_(52.73%) than APA_0.45-3 μm_(21.09%)^22^. Therefore, the phytoplankton contributed greatly to APA production. Meanwhile, the picoplankton/dissolved APA (APA_<0.45 μm_) kept a relative stable and high level (53.4% of the APA_T_). Some studies showed that the picoplankton/dissolved APA represents a significant part of the total activity. For example, Labry *et al*. reported that picoplankton/dissolved APA represented 13% to 44% of APA_T_ in the Bay of Biscay (on the French Atlantic coast)^23^. Higher proportions were recorded in the northern Red Sea (42–74%)^24^. The dissolved APase can be liberated into the environment through the lysis of dead phytoplankton cells and from cells damaged by zooplankton grazing^25^. The high values may result from physical damage of cells by water current and zooplankton grazing on phytoplankton. Nevertheless, some study found that the dissolved APA might origin from bacteria^26^. In order to elucidate the origins of dissolved APA, the dissolved alkaline phosphatase was capsulated into reverse micellar media, and it was proved that the different behaviors of dissolved phosphatase of surface and overlying water might be due to the different origins, with the former being algae and the latter being bacterial^27^. The results of Song *et al*. (2005) couldn’t identify the exact origins form, the fraction <0.45 µm contains some pico-bacteria and some pico-phytoplankton and can’t be called as the dissolved fraction. Here we changed it as the picoplankton/dissolved fraction. Besides, the positive relationships between APA and the environmental parameters that have been treated as the indexes of the productivity and trophic status, such as Chl *a*, Turb and COD, and the negative relationship between APA and SD can also indicate that the phytoplankton is the main contributor of APA.

Different algal species showed significant different secreting ability of APase. In this study, phytoplankton communities were dominated by *Bacillariophyta* in winter. *Pyrrophyta*, *Bacillariophyta* and *Chlorophyta* can easily produce extracellular phosphatase as evidenced by ELFA labeling^21^. The low APA_>3.0 μm_ during this period may result from the low algal cell density of phytoplankton and the increased concentrations of SRP. When the *Pyrrophyta* subdominated the phytoplankton community in May, the APA_>3.0 μm_ peaked. Results in some shallow eutrophic lakes revealed that the species belonging to *Pyrrophyta* were regularly phosphatase-positive, while *Bacillariophyceae* were phosphatase negative except *Aulacoseira* sp^28^. *Dinoflagellates* were poor competitors for phosphate accumulation compared to diatoms; they have to excrete much more APase than diatom to hydrolyze DOP to satisfy their P demand, even when phosphate is adequate^29^. In nutrient addition experiments, a higher percentage of *dinoflagellates* were identified with cell-specific APA than diatoms^30^. It can explain why APA_>3.0 μm_ peaked when *Pyrrophyta* subdominated the phytoplankton.It was consistent with the results in Monterey Bay that *dinoflagellates* comprised only 14% of all cells counted and accounted for 78% of APase-producing cells examined^16^. *Microcystis aeruginosa* was confirmed can also synthesize APase^31^. It can explain that as the cell density of *Cyanophyta* increased in summer and autumn, the APA_>3.0 μm_ was also prompted. The dominating of *Cyanophyta* during the summer and autumn resulted in the high amount of APA. The synchronous pattern of alkaline phosphatase activity and algal cells amount can also be found in Jialing River^32^. The higher algal cell density in midstream than in estuary can also explain why the APA_T_ was higher in midstream. It could be concluded that phytoplankton communities determined the level of APA_>3.0 μm_, which determined the significant seasonal and regional variations of APA_T_.

APA showed significant seasonal and regional variations, with lower value in inlet waters and higher value in the estuarine, and relatively low in winter and high in summer^7^. However, the distribution characteristics of APA in this study were not consistent strictly with the above mentioned. The APA_T_ fluctuated frequently from spring to autumn. Relative stable level of APA_T_ in winter can be seen in Fig. 1. This phenomenon may result from the fluctuant water level of the TGR. For the sake of flood control and hydropower, the water level in the TGR is subjected to the specific management of the TGD and is meant to seasonally fluctuate between 145 and 175 m a.s.l. It has been demonstrated that the turbulence promoted the phytoplanktonic APA and accelerated the biogeochemical cycle of P in Lake Taihu^33^. This was consistent with our results that the high APA was present during the significant water level fluctuated period from spring to autumn. Meanwhile, it has been proved that the APA increased with water temperature^34,35^. The positive relationship between WT and APA in this study (Table 1) supports the conclusion that WT determined the APA through its effects on the phytoplankton seasonally and the direct influences on APase.

## Methods

### Samples area and sites

Xiaojiang River, a tributary of the TGR, originates from Kaixian, Chongqing Municipality with a length of 180 km and watershed area of 5172.5 km^2^. It flows from north to south; entering into the TGR in Yunyang County. The distance from the estuary to the TGD is 248 km.

**Figure 8:**
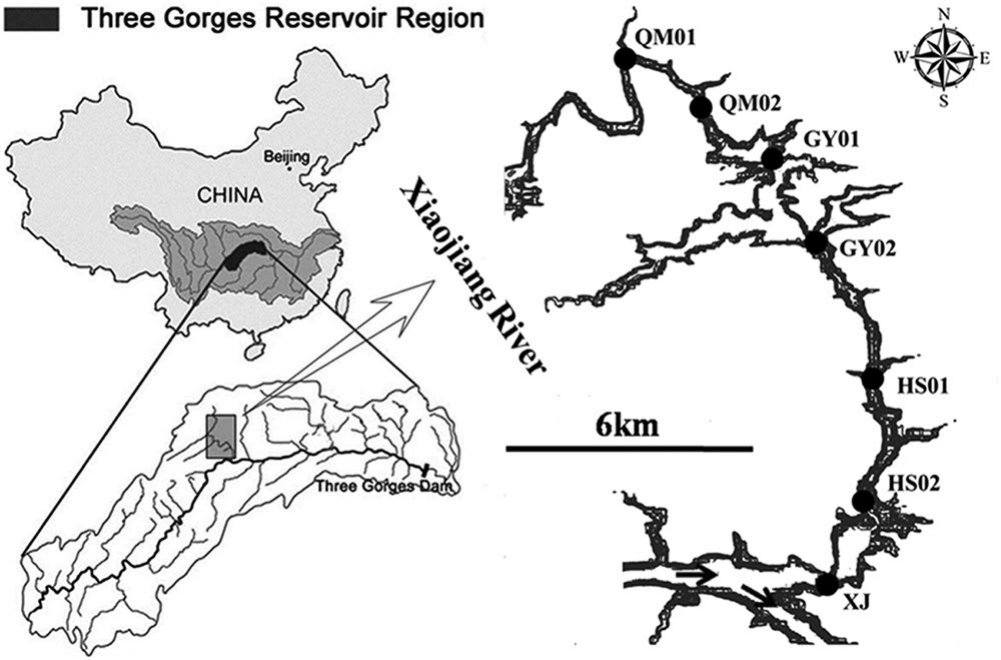
Maps of the location of the Three Gorges Reservoir Region, and the sampling sites in the Xiaojiang River (Created by Arcmap 10.0).

Water temperature (WT), pH, dissolved oxygen (DO) and conductivity (Cond.) were measured using a YSI model Professional Plus multiparameter probe (USA); Transparency (SD) was measured with a Secchi disk; and turbidity (Turb.) was measured with a WGZ-B turbidmeter (XinRui, Shanghai). Water level (WL) was recorded by GPS *in situ*. Surface water samples (0.5 m) were collected with a Van Dorn sampler at seven sampling sites (XJ, HS02, HS01, GY02, GY01, QM02, QM01) (Fig. 8) monthly from October 2013 to September 2014. All samples were run in triplicate. In order to avoid the physiological and biological parameters changed dramatically, the water samples for APA test were filtered immediately after collection and strong oscillation *in situ*, the filters were put into a portable refrigerator at 0 °C and analyzed within 24 h. All water samples for the other parameters measurement were also stored in a portable refrigerator at 0 °C after collected and tested within 24 h. Concentrations of chlorophyll *a* (Chl *a*), total phosphorus (TP), soluble reactive phosphorus (SRP), chemical oxygen demand (COD) were analyzed after samples collected within 24 h. Samples for quantitative phytoplankton analyses were fixed with neutral Lugol’s solution, and concentrated after 48 h sedimentation^36^.

### Measurement of APA

APA was measured using a modified procedure^37,38^. A total of 2 ml water samples were incubated at 37 °C for 4 h in the presence of 1 ml 0.05 M Tris-HCl buffer (pH = 8.5) and 2 ml 0.3 mM p-nitronphenylphosphate (p-NPP) as substrate, subsequently, 0.1 ml 0.1 M NaOH was added into the mixture after 4 h. The release of p-nitrophenol from p-nitronphenylphosphate was determined by absorbance at 410 nm using a spectrophotometer (TU-1810), and APA was calculated in nM·L^−1^·min^−1^. APA was determined in unfiltered water (APA_T_) and water samples filtered through 0.45 (the picoplankton/dissolved alkaline phosphatase activity, APA_<0.45 μm_) and 3.0_ μm_ membrane filters (APA_<3.0 μm_). The activity in algal fraction (APA_>3.0 μm_) and in bacterial fraction (APA_0.45-3.0 μm_) were calculated as follows: APA_>3.0 μm_ = APA_T_−APA_<3.0 μm_, APA_0.45-3.0 μm_ = APA_<3.0 μm_−APA_<0.45 μm_^39^.

### Measurement of SRP, Chl *a*, TP, COD and phytoplankton quantification

Water samples used for the Chl *a* measurement were filtered with Whatman GF/C filter, then the residuals on the filter were extracted using 90% acetone solution in the darkroom for 24 h at 4 °C, and Chl *a* was analyzed spectrophotometrically. The concentrations of SRP were measured after all water samples were filtered through pre-washed filters (Whatman GF/C, glass microfiber filters).The concentrations of SRP, total phosphorus (TP) and chemical oxygen demand (COD) were analyzed according to the standard methods^40^. Phytoplankton was quantified at 400 × magnification with a light microscope (OLYMPUS BX41). The identification of phytoplankton species is according to Hu and Wei^41^.

### Statistical analysis

Statistical analysis was carried out using the SPSS 13.0 package. Variance analysis (one-way ANOVA) was used to compare the means of APA in different seasons and sampling sites. Normal distribution of the data was ensured by visual inspection of Q–Q plots, and Levene’s test was used to check for homogeneity of variances before ANOVA. Non-parametric correlation (Spearman) analyses were employed for determining relationships among APA_<0.45 μm_, APA_0.45-3 μm_, APA_>3.0 μm_, APA_T_ and the environmental factors. Detrended correspondence analysis (DCA) of the size-fractionated APA and environmental data was performed using CANOCO version 4.5 to determine whether linear or unimodal ordination methods should be applied^42^. Before the analysis, the abiotic and biological data were transformed by log(x + 1). We calculated an unconstrained ordination through the DCA. Using detrending by segments and Hill’s scaling, the length of the longest axis estimated the beta diversity in the data set. Moreover, the value of the data set implies that it is appropriate to use the Redundancy analysis (RDA) method. RDA was performed to get an approximate ordering of the size-fractionated APA’s optima for environmental variables. The significance of canonical axes and environmental variables to explain the variance of the size-fractionated APA was tested using Monte Carlo simulations with 499 permutations^43,44^.

### Data Availability

All data analyzed during this study are included in this published article.
